# Glances at the Hospitals

**Published:** 1903-09-12

**Authors:** 


### GLANCES AT THE HOSPITALS.

### MANCHESTER SOUTHERN HOSPITAL FOR WOMEN AND CHILDREN.

The income was £3,422 and the expenditure exceeded £4,005, leaving a deficiency of £582, which, added to that of the previous year, makes an adverse balance of £1,907. The committee point out the urgent need of increased subscriptions and donations, and that owing to the lack of funds during the year some of the accommodation has had to be closed. It is much to be desired that this will not be allowed to continue long. Manchester should not hesitate to provide the amount of money which the committee require to properly carry on the work of the hospital. As regards the arrangement of the report there is much to be desired. In some cases the information given is hopelessly deficient. It is useless stating that 26 abdominal sections were performed unless the results are also stated, and no such particulars are inserted in the tables. On page 11 it is stated that there were 199 cases in the maternity department, and a table follows stating there were 67 abnormal cases. Next to this is a statement giving the cause of death in eight cases, but whether these deaths refer to the total of 199 in-patients or to the 67 abnormal cases is not apparent.

### EAST SUFFOLK AND IPSWICH HOSPITAL.

The receipts amounted to £5,219 and the expenditure to £6,209, showing a balance of £990 on the wrong side. It is satisfactory to find that the workpeople's own subscriptions have again increased and now amount to £646. At the beginning of the year the hospital contained 91 patients and 992 were admitted since, making a total of 1,083 under treatment. Of these 558 were discharged cured; 252 were discharged much improved; 116 were discharged for other reasons; 51 died; and 106 remained in the hospital. The average cost per in-patient was £4 13s. 1d., and the average cost per week was 19s. 4½d. The average duration in the wards was 29 days. We did not see the medical and surgical tables usually given in some form or other in these hospital reports but there is a table of operations performed during the year, and it is correctly arranged, and gives the essential information regarding the various operations which reached a total of 765. There are also tables relating to the eye department; but these do not show the results of the operations. There is no anæsthetic table.

### NEWTON ABBOT HOSPITAL.

Here the income amounted to £1,380 and the expenditure to £1,187, showing a balance of £193 on the right side—a state of finance so rare in hospitals that it deserves being chronicled when found. The weekly payments of the ordinary patients came to £93, and those of the private patients, of whom there were 7, to £114. The number of patients admitted during the year was 221, and the number treated in the casualty and out-patients' department was 764. A register of in-patients is given which takes the place of the usual medical and surgical tables. This register gives the date of admission, age of patient and his residence, and occupation, the disease from which he suffered, the medical officer attending him, and the result. For purposes of reference to any particular case this register would be very useful, but we think it would be better not to publish it and let the usual tables be substituted in future reports. Comparison with other hospitals could then be more easily made.

### HULL ROYAL INFIRMARY.

The total ordinary receipts amounted to £10,651, and the total ordinary expenditure to £10,342. Legacies for investment amounted to £4,680. The infirmary is being enlarged, and it is said that about £40,000 will be required for this purpose. The working men's subscriptions to the ordinary expenditure shows a satisfactory increase, having risen from £1,851 in 1901 to £2,292 in 1902. The average cost of each patient was £3 2s. 8d.; the cost of each patient per week was 17s. 1d. The average cost per bed occupied was £50 19s. The average number of beds occupied was 168, and the average stay in the hospital was nearly 26 days. The medical and surgical report is the most meagre we have yet met in any hospital approaching that of Hull in size or importance. There are no tables given, and the information vouchsafed to us is comprised in a short summary of a page and a half. From it we learn that 2,302 in-patients were treated during the year; that there were 243 deaths; that the number of major operations was 765; that 200 of these were abdominal sections; that in the *x*-ray department 375 examinations were made; and that in the electro-therapeutic department 49 cases were treated. It would surely have been time well spent to tabulate the results, more especially as regards the 200 abdominal sections and the 49 cases subjected to the electro-therapeutic treatment.

### ABERDEEN HOSPITAL FOR SICK CHILDREN.

The total ordinary income amounted to £1,876, and the total ordinary expenditure to £3,230, showing a deficit on this account of £1,354, but legacies amounting to £200 reduced the adverse balance. Putting the last year's deficit with this it is seen that the hospital is £2,524 in debt; and, therefore, the Committee appeal to the sympathy which all classes of the community feel towards poor sick children. We hope that this appeal may bring in the wished-for subscriptions; but in the meantime it would surely be desirable to lessen the expenditure in some way rather than incur burdens for other years to wipe off. We know that it is difficult for committees to close any of the wards when patients are knocking for admission; but if the inhabitants of the district will not subscribe the requisite amount of money, there is no other course open to them in the end. Mr. David Pearson deserves mention for having given £600 to endow a cot, and Mrs. Stephen Wilson left £1,000 for a similar purpose. The tables are admirably drawn out, and the results can be followed at a glance. There were 30 cases of diphtheria, and of these 26 were cured, 3 died, and 1 remained under treatment. Of 37 cases of pneumonia, 16 recovered, 1 improved, 1 left before treatment was finished, 13 died, and 6 remained in the hospital. The total number of operations was 334. Of these 210 recovered, 103 relieved, 9 were removed, and 12 died. There is no anæsthetic table.

### THE PETERBOROUGH INFIRMARY.

The total income amounted to £2,227 and the total expenditure to £2,193. During the year 612 patients were treated in the Infirmary, and of these 22 were private patients. The medical and surgical tables are very well arranged, and even the operation table shows the number cured, unrelieved, died, and remaining under treatment. There is also a column for "remarks," but no anæsthetic table is printed. The total number of operations was 448.

